# *Myristica Fragrans* Houtt Extract Attenuates Neuronal Loss and Glial Activation in Pentylenetetrazol-Induced Kindling Model

**DOI:** 10.22037/ijpr.2019.1100670

**Published:** 2019

**Authors:** Davoud Ghorbanian, Maryam Ghasemi-Kasman, Mona Hashemian, Elaheh Gorji, Mohammad Gol, Farideh Feizi, Sohrab Kazemi, Manouchehr Ashrafpour, Ali Akbar Moghadamnia

**Affiliations:** a *Student Research Committee, Babol University of Medical Sciences, Babol, Iran. *; b *Cellular and Molecular Biology Research Center, Health Research Institute, Babol University of Medical Sciences, Babol, Iran. *; c *Neuroscience Research Center, Health Research Institute, Babol University of Medical Sciences, Babol, Iran.*; d *Department of Physiology, Faculty of Medicine, Babol University of Medical Sciences, Babol, Iran.*; e *Department of Pharmacology, Faculty of Medicine, Babol University of Medical Sciences, Babol, Iran.*

**Keywords:** Epilepsy, Pentylenetetrazol, Nutmeg extract, Neuroprotection, Glial activation

## Abstract

Inflammatory reactions are closely associated with the development and progression of epilepsy. It has been shown that inhibition of pro-inflammatory cytokines, which are released from activated astrocytes and microglia, are considered to be an effective therapeutic approach for the treatment of epileptic disorders. Regarding the anti-inflammatory effects of nutmeg (*Myristica fragrans *Houtt), the present study was designed to investigate whether the nutmeg ethanolic extract could exert anticonvulsant and inhibitory effects on glial activation in pentylenetetrazol (PTZ)-induced mice model of kindling. Ethanolic extract of nutmeg was administrated intraperitoneally (i.p.) 1 hour before PTZ injection or one week before PTZ as a separate group, to become fully-kindled. The chemical components of nutmeg extract were analyzed by gas chromatography mass spectrometry (GC-MS). Immunostaining against neuronal and glial markers was performed on hippocampus sections. GC-MS data indicated that the main components of nutmeg extract are myristic acid (39.93%), elemicin (22.16%) and myristicin (11.17%). Behavioral studies showed that pre-treatment of nutmeg extract effectively reduced seizures behavior, decreased cell death, and ameliorated glial activation that is followed by PTZ administration. In conclusion, nutmeg extract might be regarded as a useful supplementary agent in epilepsy treatment through its attenuation of neuronal loss and glial activation.

## Introduction

Abnormal electrical activity in epileptic seizures results from an imbalance between excitation and inhibition (1). In addition, inflammatory processes have an important role in the pathogenesis of epilepsy (2). Experimental and clinical evidences have suggested that pro-inflammatory factors and cytokines are overexpressed in epileptic conditions and these factors can enhance brain excitability and alter microglia and astrocyte functioning (3). Neuro-inflammation results primarily from the activation of glial cells including astrocytes and microglia. Activated glial cells secrete variety of inflammatory cytokines (3-5). These inflammatory cytokines play an important role in the progression of epilepsy (2). Previous studies indicated that some anti-inflammatory drugs reduce seizure behavior in experimental models and clinical cases of epilepsy (2, 6, 7). Despite the availability of several anti-epileptic drugs, these medications are effective only in about 40% of epileptic patients (8). In addition, anti-epileptic drugs have different side effects (9). Based on these limitations, a demand for finding new types of anticonvulsant drugs exist. Recent studies have demonstrated that herbal medicines (phytomedicine) might be effective in the treatment of some epileptic cases (10). Nutmeg (*Myristica fragrans *Houtt.) is widely being used as a spice in the food industry worldwide. Nutmeg, or its effective substances, have been shown to possess beneficial effects in the treatment of several diseases such as rheumatoid arthritis, diarrhea, mouth sore, and insomnia (11). Numerous evidences have demonstrated that nutmeg has several pharmacological functions such as anti-bacterial, anti-microbial, anti-fungal (12), and antioxidant effects (13). Additionally, the anti-inflammatory effect of nutmeg has been evaluated both *in-vitro *and *in-vivo *(14-16). It has been shown that nutmeg, or its active compounds, effectively alleviate inflammation by inhibiting various inflammatory factors (14-16). The pentylentetrazol (PTZ)-induced kindling model is one of the most reliable models for studying the epileptogenesis process. Repetitive administration of PTZ at sub-convulsive dose leads to the induction of chemical kindling model in animals. The effects of novel anti-epileptic compounds can be evaluated by either applying them before the initiation of kindling (pre-kindling phase) or after becoming fully-kindled (post-kindling phase) (17). 

The present study was designed to elucidate the effects of nutmeg extract on the development of kindling and neuronal death following PTZ administration, based on the role of inflammation on epilepsy and the anti-inflammatory properties of nutmeg extract. In addition, glial activation was evaluated in animals under nutmeg extract treatment. 

## Experimental

*Preparation of nutmeg extract*

Nutmeg seeds purchased from our local market in Babol and approved by the department of Pharmacology, Babol University of Medical Sciences (herbarium number: 94/301). Preparation of extract was performed based on our previous report (18). In brief, 300 grams of nutmeg seeds was powdered with a mechanical grinder. After addition of 500 mL absolute ethanol, the powder was shaken for 72 h. The supernatant solvent was removed using a rotary evaporator (in a vacuum, temperature below 45 °C). To completely evaporate the solvent, the prepared extract was placed into a plate in the oven. 

*Gas chromatography-mass spectrometry analysis of nutmeg extract*

Nutmeg extract was analyzed using Agilent 7890B gas chromatograph (Agilent Technologies, Palo Alto, CA, USA) equipped with 5977B mass spectrometer. Helium gas, with a flow rate of 1 mL/min, was used as the carrier gas. Volatile components were separated by an HP-5MS capillary column (polydimethylsiloxane5% diphenyl, 30 m×0.32 mm×0.25 mm, Hewlett Packard). The temperature was set to rise from 40 °C (1 min) to 220 °C at 5 °C/min, with a 1 min hold at the end. The compounds were identified by comparing their mass spectra and elution order with those obtained from the National Institute of Standards and Technology (NIST 08) and Wiley 7n library data of the GC-MS system.

*Animals*

Male NMRI mice (20-30 g) were obtained from the animal house of Babol University of Medical Sciences (Babol, Iran). Animals were kept in 12 h light/dark cycle with free access to food and water. All experimental procedures were approved by the local ethical committee of Babol University of Medical Sciences, which is in according to international guidelines on the use of laboratory animals. 

*PTZ-induced kindling model*

Chemical kindling model was induced as described in our previous study (19). Briefly, PTZ was administrated intraperitoneally (i.p) at dose of 36.5 mg/kg every 48 h for 18 consecutive days. Animals were monitored after each PTZ injection for 20 min and seizure behavior was scored as follows: Stage 0, no response; Stage 1, ear and facial twitching; Stage 2, myoclonic jerks (MJ), Stage 3, clonic forelimb convulsions; Stage 4, generalized clonic seizures along with turning to a side position, and Stage 5, generalized clonic-tonic seizures (GCTS) or death (20). In this study, latency to the onset of MJ (S2 latency), duration of GCTS (S4 & S5 duration) and maximum seizure stage were assessed after each PTZ injection. 

*Experiment design*

Forty-two male NMRI mice were randomly divided to 6 experimental groups (n = 7) as follows: Saline +PTZ, which received i.p. injections of sterile saline containing 0.1% Tween-80 as nutmeg extract vehicle, 1 h before each PTZ injection; nutmeg + PTZ group, where nutmeg extract at 50 or 100 mg/kg doses was administrated i.p. 1 h before each PTZ injection. In another approach, animals received nutmeg extract pre-treatment for 1 week and continued as shots 1 h before each PTZ injection. Groups had the same amount of subjects as the first approach. Animals were sacrificed after evaluating seizure behavior and electrophysiological recordings, and the level of glial activation and neuronal density were assessed using immunostaining and nissl staining, respectively. 

*Electroencephalogram recording*

After the 6th PTZ injection, electroencephalogram recordings were carried out based on a previous report by Gol *et al*., (21). Animals were anesthetized with ketamine (100 mg/kg) and xylazine (10 mg/kg) and fixed in a stereotaxic apparatus (Stoleting, USA). Two monopolar electrodes were implanted into the frontal and parietal lobes of the cortex and fixed with dental cement. Electroencephalogram (EEG) recording was performed in freely moving animals for 10 min after the last injection of PTZ (injection 9). EEG signal was band pass filtered 1-100 Hz and amplified 500 times (D3111 Data Acquisition, ScienceBeam Co., Tehran, Iran). 

*Immunostaining*

After deep anesthesia with ketamine (70 mg/kg) and xylazine (10 mg/kg), animals were transcardially perfused with phosphate buffer saline (PBS) and paraformaldehyde (PFA) 4%. Brain samples were removed and submerged in PFA for 12-16 h at 4 °C. Samples were dehydrated using 30 % sucrose. Brain tissues were embedded in optimal cutting temperature (OCT) compound and coronal sections (6 µm) from the dorsal part of the hippocampus (bregma from -1.46 mm to -2.30 mm) with 120 µm intervals were prepared using cryostat instrument (MICROM HM 525, Thermo Scientific). Immunostaining was performed according to our previous report (20). Briefly, after washing with PBS, sections were permeablized by Triton X100 0.3 % for 20 min. None-specific bindings were blocked using blocking solution (normal goat serum (NGS) 10% in PBS) for 1 h. Then, sections were incubated with primary antibodies including rabbit anti-GFAP (1:400, Dako, Denmark), rabbit anti-Iba1 (1:200, Wako, USA) or rabbit anti-NeuN (1:500, Abcam, Cambridge, UK) at 4 °C overnight. Then, tissue sections were incubated with appropriate anti-rabbit IgG conjugated with FITC (1:100, Abcam, Cambridge, UK) and anti-rabbit IgG conjugated with Alexa 594 (1:1000, Abcam, Cambridge, UK) for 1 h at room temperature. Nuclear staining was performed by 4′,6-diamidino-2-phenylindole (DAPI). Tissue sections were evaluated under fluorescence microscope (Olympus IX71, Japan) and images were captured using DP-72 camera (Olympus, Japan). Quantification of histological results were performed using image J software (version 1.42 V, NIH, USA) as we have described previously (19). Three sections from each slide and 3 slides from each animal were used for histological analysis. The number of animals for each group was 3 mice (27 sections for each experimental group).

**Figure 1:**
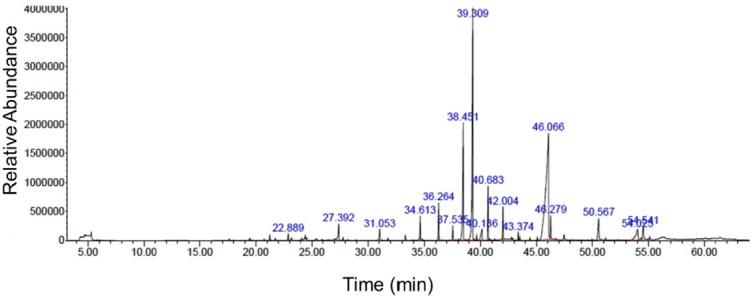
GC-MS chromatogram of nutmeg ethanolic extract. GC-MS analysis showed the different compounds which were present in the extract

**Figure 2:**
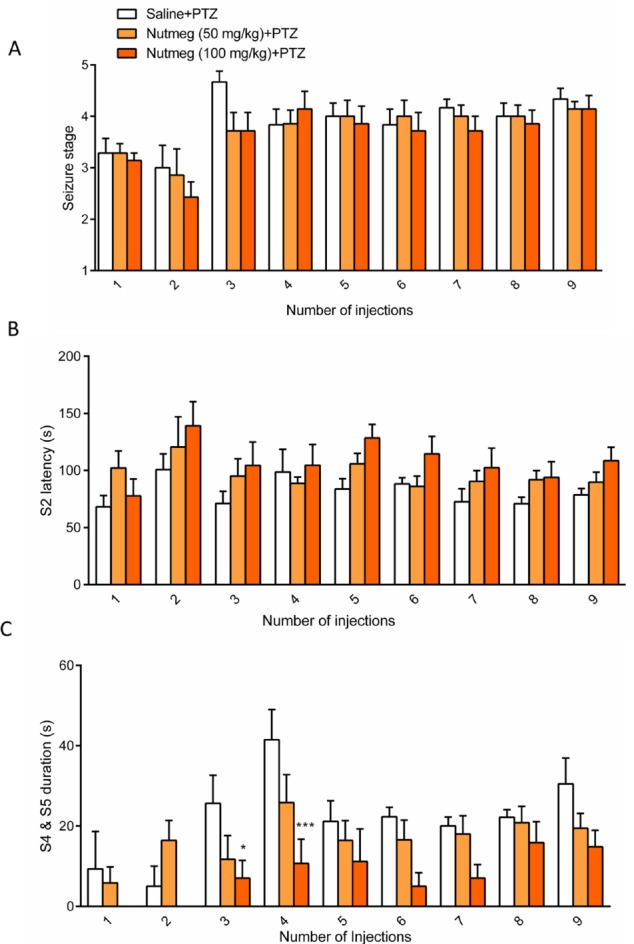
Effect of nutmeg administration on seizure behavior

**Figure 3:**
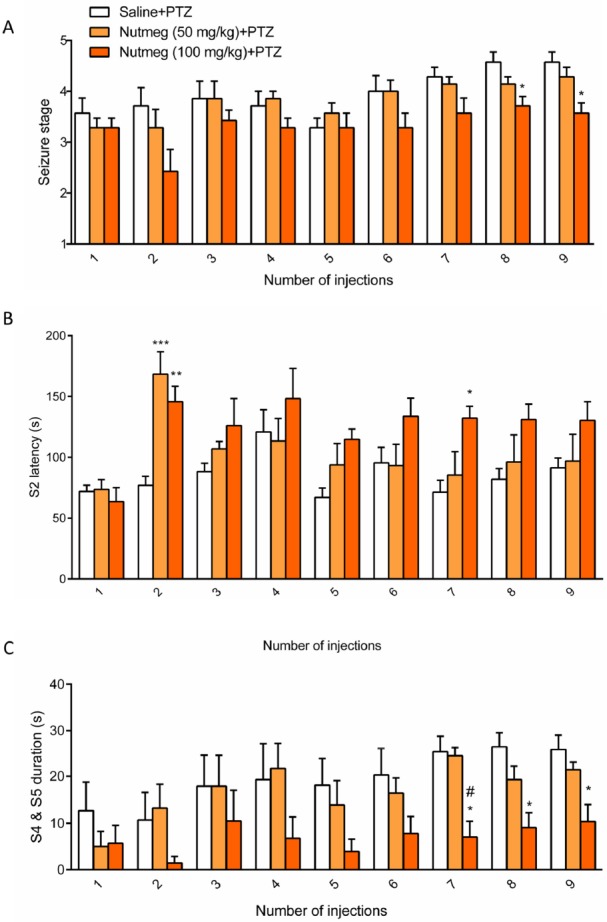
Effect of nutmeg pre-treatment on and seizures behavior

Effect of nutmeg pre-treatment on seizure stage (A), latency to the onset of MJ (S2 latency) (B), and duration of GCTS (C). Maximum seizure stage data was analyzed by Kruskal-Wallis non-parametric test followed by Dunn′s post-test and S2 latency/GCTS was assessed using two way-ANOVA, followed by Bonferroni post-hoc test.*p < 0.05 and ** *p *< 0.01 and ***p < 0.001 compared to saline +PTZ; # *p*

< 0.05 compared to nutmeg (50 mg/kg) +PTZ, n = 7.

**Figure 4:**
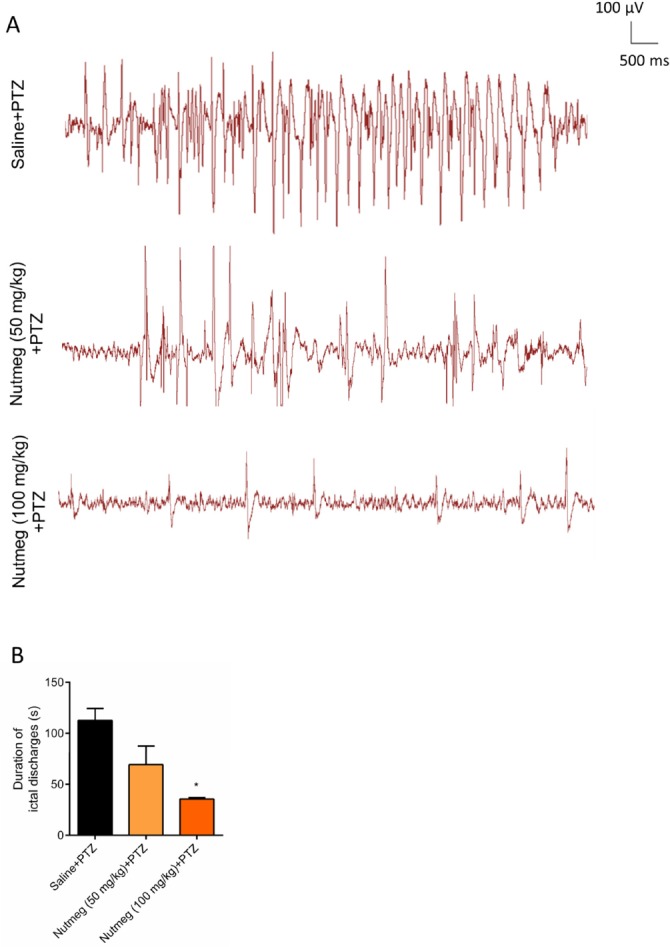
Effect of nutmeg extract on duration of ictal discharges in PTZ-induced kindling model

- Sample records of EEG recording right after the last PTZ injection. Scale bar: 100 µV, 500 ms.
- Analysis of EEG recording using One-way ANOVA, followed by Tukey post-test showed that nutmeg pre-treatment at 100 mg/kg dose reduced the duration of ictal discharges compared to saline + PTZ. * *p *< 0.05, n = 3.

**Figure 5:**
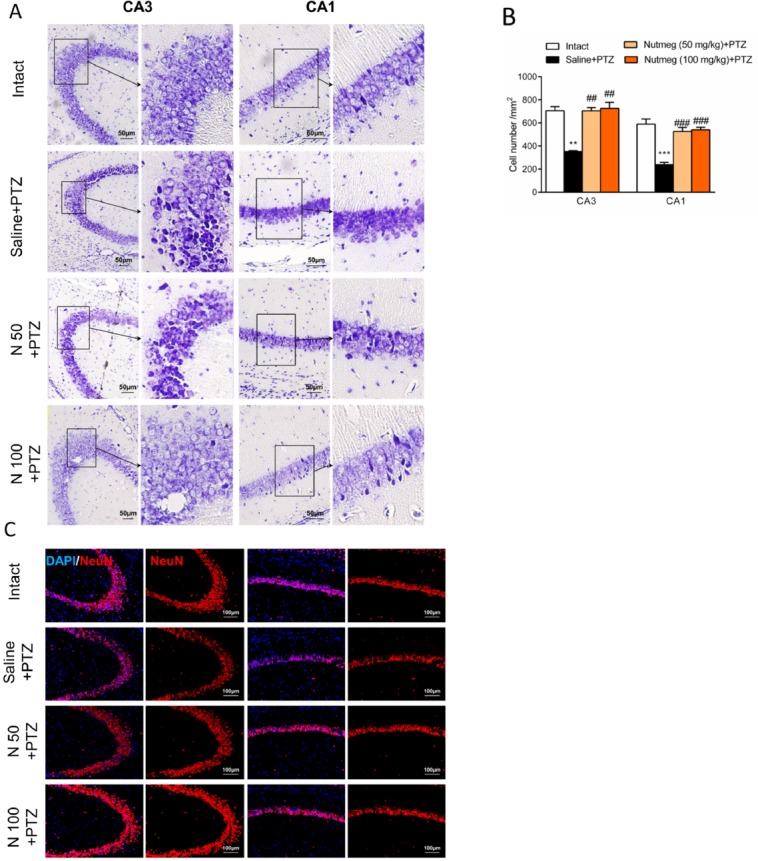
Effect of nutmeg extract pre-treatment on hippocampal cell density following PTZ injection

- Nissl staining was performed on dorsal hippocampus in PTZ-induced kindling model.
- Cell counting in CA3 and CA1 regions of hippocampus indicated that nutmeg pre-treatment at both doses significantly reduced cell death compared to saline. One way ANOVA, followed by Tukey post-test was used for histological data analysis. **p < 0.01 and ***p <

0.001 compared to intact; ## *p *< 0.01 and ### *p *< 0.001 compared to saline + PTZ. Scale bar: 50μm, n = 3.

C) Immunostaining against NeuN, as a mature neuronal marker, indicated that the number of neurons were higher in nutmeg receiving

groups compared to saline. Scale bar: 50μm, n = 3.

**Figure 6:**
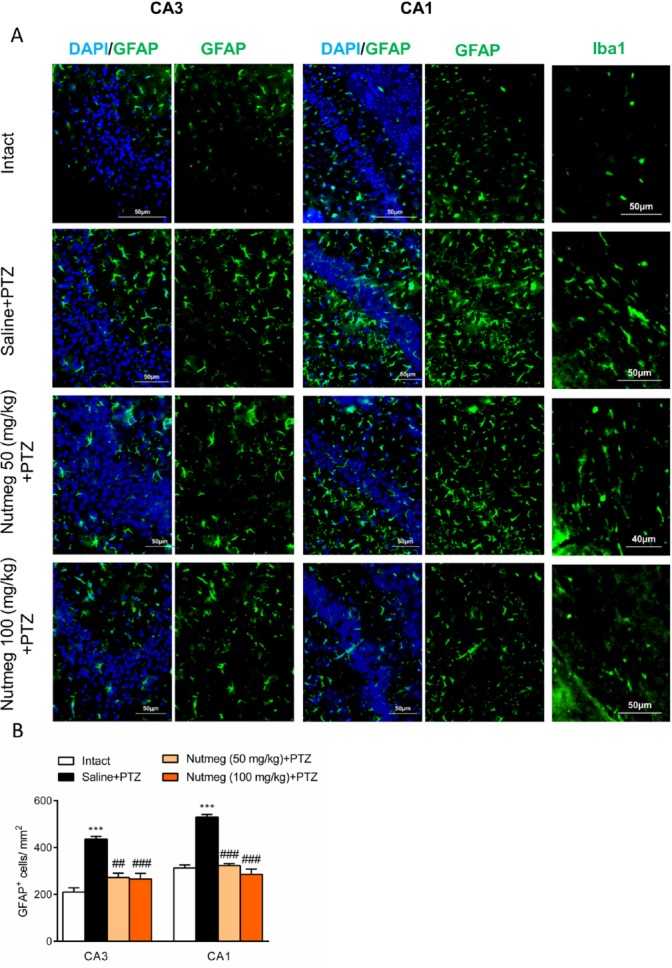
Effect of nutmeg pre-treatment on glial activation

- Immunostaining against GFAP, as an astrocyte, and Iba1, as a microglia marker, on hippocampus sections following PTZ injection.
- Quantification of GFAP immunostaining results. Nutmeg extract pre-treatment ameliorated astrocyte activation in both CA3 and CA1 regions of hippocampus in PTZ receiving animals. One way ANOVA, followed by Tukey post-test was used for histological data analysis. ***p <0.001compared to intact group; ## *p *< 0.01 and ### *p *< 0.001 compared to saline + PTZ, Scale bar: 50μm, n = 3.

**Figure.7:**
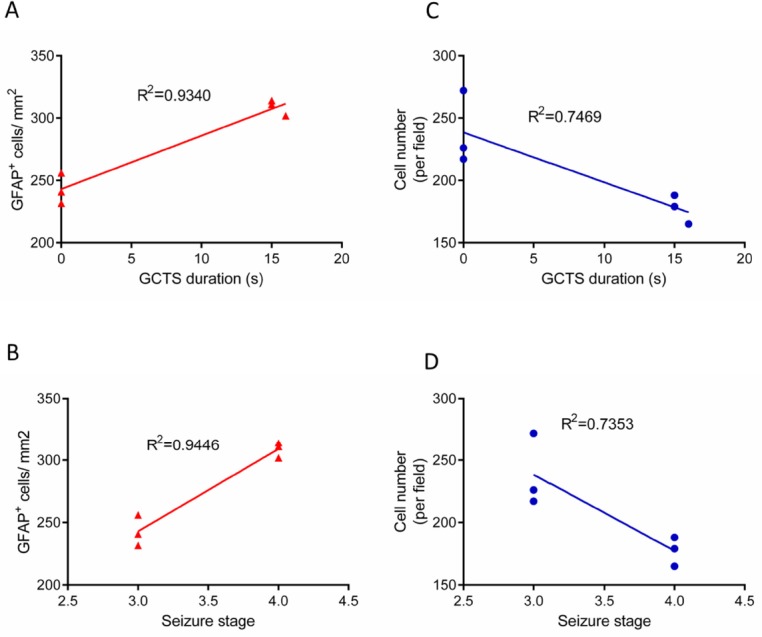
Linear regression analysis between seizure behavior and astrogliosis/cell density in hippocampus

- Correlation between GCTS duration and GFAP positive cells in hippocampus of PTZ receiving animals.
- Correlation between duration of GCTS and cell density.
- Correlation between seizures stage and GFAP-immunoreactive cell counts in dorsal hippocampus.
- Correlation between seizures stage and cell density in hippocampus.

**Table 1:** Nutmeg chemical compounds identified using GC-MS

**Retention time (min)**	**Name of compounds**	**Area (%)**
22.888	gamma-Terpinene	0.48
27.389	4-Terpinol	1.79
31.054	Benzene 1,2- (methyllenedioxy)-4-propenyl	1.02
34.611	1-cyclopropyl-3,4-dimethyoxyeugenol	1.76
36.270	lsoeugenol	2.59
37.538	Cis-Methyl isoeugenol	0.97
38.449	Myristicin	11.17
39.306	Elemicin	22.16
40.141	Lauric acid	1.90
40.683	Methyl eugenol	3.80
42.006	Trans-lsoelemicin	2.14
43.372	Phenol, 2, 6-dimethoxy-4- (2-propeyl)	0.46
46.062	Myristic acid	39.93
46.279	Myristic acid ethyl ester	1.57
50.562	Palmitic acid	2.86
54.021	1- Hydroxy-o-methylsterigmatocystin	3.44
54.542	Oleic acid	1.96

*Nissl staining*

As mentioned for immunostaining experiments, brain tissues were removed and post-fixed in PFA overnight. Dehydration was done by graded alcohols and tissues were embedded in paraffin. Serial coronal sections (6 µm) were prepared using a microtome (Leica RM2135, Germany). Briefly, after deparaffizination and rehydration with alcohol, nissl bodies in perikaryon were stained using creysl violet (Merck, Germany) for 4 min. Sections were washed with running water, dehydrated, and mounted with entellan (Merck Chemicals, Germany). Images were collected from CA1 and CA3 regions and quantification of nissl staining data was carried out as we have explained for immunostaining procedures. 

*Statistical analysis*

Maximum seizure stages were analyzed by Kruskal-Wallis non-parametric test followed by Dunn′s post-test. Latency to the onset of MJ and GCTS data were assessed using two way-ANOVA, followed by Bonferroni post-hoc test. Histological results were analyzed by one-way ANOVA, followed by Tukey post-test. Linear regression analysis was used to determine the correlation between behavioral and histological results.* p* values < 0.05 were considered statistically significant.

## Results

*Myristic acid, elemicin and myristicin as major chemical components of nutmeg extract*

Chemical compositions of nutmeg extract were analyzed by GC-MS technique. GC-MS analysis results identified 17 different components representing 100% of the total extract (Table 1). The major compounds of nutmeg extract were myristic acid (39.93%), elemicin (22.16%), and myristicin (11.17%) (Figure1). Other important components are isoeugenol (2.59%), methyl eugenol (3.80%), 1-Hydroxy-o-methylsterigmatocystin (3.44%), trans-Isoelemicin (2.14%) and palmitic acid (2.86%).

*Pre-treatment of nutmeg extract decreased seizure behavior in PTZ- induced kindling model*

To assess the effect of nutmeg on seizure behavior, alcoholic extracts of nutmeg (50 or 100 mg/kg) were administrated 1 h before every PTZ injection. Behavioral analyses revealed no significant difference in seizure stage between control and nutmeg receiving groups (Figure 2A). Additionally, in animals which received the nutmeg extract, latency to the onset of MJ (S2 latency) was not significantly affected compared to saline group (Figure 2B). Administration of nutmeg extract did not reduce the duration of GCTS and just a significant effect of nutmeg extract was seen at 100 mg/kg dose compared to saline + PTZ at injection 3 (25.667±6.979 in saline +PTZ and 7 ± 4.457 s in nutmeg (100 mg/kg)+PTZ, ^* ^*p* < 0.05) and 4 (41.500±7.478 s in saline +PTZ and 10.667 ± 6.075 s in nutmeg (100 mg/kg) + PTZ, ^***^ *p* < 0.001) of PTZ (Figure 2C).

To assess the effect of nutmeg pre-treatment on seizure behavior, nutmeg extract was administrated 1 week before PTZ injection and continued until the end of experiment. Behavioral evaluation indicated that the 100 mg/kg dose of nutmeg significantly decreased the mean seizure stage compared to saline + PTZ at injection 8 (4.571 ± 0.20 in saline + PTZ and 3.714±0.1844 in nutmeg (100 mg/kg) +PTZ, ^*^ *p* < 0.005) and 9 (4.571 ± 0.2020 in saline + PTZ and 3.571 ± 0.2020 in nutmeg (100 mg/kg) +PTZ, ^*^ *p* < 0.05) of PTZ (Figure.3A). Additionally, a significant increase in latency of MJ was observed in animals under nutmeg extract treatment at 100 mg/kg dose compared to saline at injections 2 and 7 (Figure. 3B). Furthermore, pre-treatment of animals with dose of 50 mg/kg could also significantly increase the MJ latency at injection 2 of PTZ (^***^p < 0.001) (Figure. 3B). For the latency of MJ, two way ANOVA revealed a significant main effect of nutmeg extract treatment [F _(2, 18)_ =5.625, 

*p *=0.0127], time [F (8,144) = 6.071, *p *< 0.0001], as well as nutmeg treatment × time interaction [F _(16,144)_ = 1.948, *p* = 0.0205]. Interestingly, the duration of GCTS effectively reduced in animals under pre-treatment of higher dose of nutmeg extract compared to saline + PTZ at injections 7, 8 and 9 of PTZ (^*^p < 0.05) (Figure.3C). In addition, we could find a significant difference in GCTS duration between nutmeg experimental groups at injection 7 of PTZ (^#^ *p* < 0.05) (Figure 3C). For the duration of GCTS, two way ANOVA revealed a significant main effect of nutmeg treatment [F _(2, 16)_ = 6.458, *p* = 0.0088], time [F _(8, 128)_ = 6.039, *p* < 0.0001], but not a significant effect of nutmeg treatment × time 

[F _(16, 128)_ = 1.159, *p* = 0.3095].

In order to evaluate the effect of nutmeg pre-treatment on duration of ictal discharges, electrophysiological recording (EEG) was performed after the last injection of PTZ. Figure 4A is a sample of EEG recordings in PTZ receiving animals. Analysis of EEG recording data indicated that there was robust ictal discharges in fully-kindled animals receiving vehicle. In contrast to vehicle group, pre-treatment of animals with high dose of nutmeg extract significantly reduced the duration of ictal discharges following PTZ injection (112.6 ± 11.70 s in saline + PTZ and 35.52 ± 1.400 s in nutmeg (100 mg/kg) + PTZ (^*^ *p* < 0.05) (Figure 4B).

*Nutmeg pre-treatment reduces cell death in hippocampus following PTZ injection*

It has been shown that PTZ-induced seizures cause a remarkable neuronal loss in different brain regions, especially in the hippocampus (19). In order to evaluate the effect of nutmeg pre-treatment on hippocampal cell density, nissl staining was performed on brain sections. Histological staining results and its quantification showed that a high percentage of cell death was observed in CA3 (705.3 ± 34.78 in intact group and 352.1 ±7.622 in saline + PTZ, ^**^ *p* < 0.01) and CA1 regions (589.4 ± 44.30 in intact and 238.8 ± 19.31 in saline +PTZ, ^***^ *p* < 0.001) of PTZ receiving animals which were treated with saline compared to intact group. In contrast to vehicle group, both doses of nutmeg extract significantly attenuated cell death in CA3 (115.5 ± 2.5 in saline +PTZ, 231 ± 9 in nutmeg (50 mg/kg)+PTZ , ^##^ *p* < 0.01 and 238 ± 17.03 in nutmeg (100 mg/kg)+PTZ, ^##^ *p* < 0.01) and CA1 regions of hippocampus (78.33 ± 6.333 in saline +PTZ, 172.7±11.26 in nutmeg (50 mg/kg) + PTZ, ^###^ *p* < 0.001; 177.3 ± 6.692 in nutmeg (100 mg/kg) +PTZ, ^###^ *p* < 0.001) (Figure 5A-B). To further confirm the nissl staining data, NeuN antibody was applied on the dorsal hippocampus, as a mature neuronal marker. Immunostaining data indicated that the number of NeuN positive cells in CA3 and CA1 regions of hippocampus were higher in animals under treatment of nutmeg extract compared to saline + PTZ (Figure 5C).

*Nutmeg extract pre-treatment reduces glial activation of hippocampus following PTZ injection*

Glial activation in hippocampus drastically increases following repetitive administration of PTZ (22). In order to characterize the effect of nutmeg administration on glial activation in PTZ-induced kindling model, immunostaining against GFAP, as an astrocyte marker, was performed on brain sections of the dorsal hippocampus. Immunostaining results and its quantification revealed that the number of GFAP expressing cells increased in fully kindled animals which received saline in comparison to intact animals (CA1: 210.4 ± 17.86 in intact, 436±11.54, ^***^ *p* < 0.001; CA3: 313 ± 12.97 in intact, 530.5 ± 10.99 in saline +PTZ, ^***^ *p* < 0.001). Pre-treatment with nutmeg extract at both doses significantly reduced the level of astrocytes activation in both CA3 (436 ± 11.54 in saline + PTZ, 272.4 ± 18.49 in nutmeg (50 mg/kg)+PTZ, ^##^ *p* < 0.01; 266.3 ± 23.44 in nutmeg (100 mg/kg)+PTZ, ^###^ *p* < 0.01) and CA1 regions of hippocampus (530.5 ± 10.99 in saline +PTZ, 323.2 ± 8.066 in nutmeg (50 mg/kg) + PTZ, ^###^ *p* < 0.001; 285.6 ± 22.63 in nutmeg (100 mg/kg)+PTZ, ^### ^*p* < 0.001) (Figure. 6A-B). In consistence with GFAP staining, immunostaining against Iba1, as a microglia marker, also indicated that Iba1expression remarkably increased in fully-kindled animals and nutmeg administration decreased the activation of microglia in the hippocampus following PTZ injections (Figure 6A-B).

*Correlation between seizure behavior and histological results*

In order to determine the relationship between seizure behavior, that is, GCTS duration/ seizures stage and GFAP immunostaining results, linear correlation analysis was performed. Linear regression analysis demonstrated that the duration of GCTS and maximum seizures stage were strongly correlated with the level of astrocyte activation in hippocampus of PTZ receiving animals (R^2^=0.9340, R^2^=0.9446, respectively) (Figure 7A-B). Furthermore, the correlation between seizure behavior and cell density was determined. Regression analysis data indicated that GCTS and seizures stage were correlated to a lesser extent with hippocampus cell density (R^2^=0.7469, R^2^=0.7353) (Figure 7C-D).

## Discussion

Accumulating clinical and experimental evidences strongly support the significant role of inflammatory factors in initiation or progression of epilepsy disease (2). Findings of the present study indicate that pre-treatment of nutmeg extract decreased seizure behavior in PTZ receiving animals. Furthermore, the level of glial activation and neuronal loss were attenuated following PTZ application. Previous study by Sonavane *et al*., showed that a single injection of *Myristica fragrans* seed extract increased the latency of MJ, and convulsions were completely inhibited in acute seizure model. It seems that the anticonvulsant effect of nutmeg extract might be partly mediated via the alleviation of central dopaminergic activity (23). Additionally, another report by Wahab *et al*., demonstrated the anticonvulsant activities of nutmeg oil in animal models of seizure. It has been shown that nutmeg oil exhibits a rapid onset and short duration of anticonvulsant effect (24). In contrast to these reports, we did not observe any remarkable effects on seizure behavior when nutmeg extract was administrated 1 h before each PTZ injection. This difference may be due to PTZ dosage, type of extract, and time of nutmeg application. In a study conducted by Sonavane *et al*., PTZ was administrated at convulsive dose (80 mg/kg) to induce acute seizure model in animals, while in our study, PTZ was administrated at sub-convulsive dose (36.5 mg/kg) to develop kindling model. Additionally, in the former study, nutmeg extract was administrated 30 min before PTZ injection (23). To evaluate the anti-seizure effects of nutmeg extract, we used another approach in which the nutmeg extract was administrated for a week before the injection of PTZ. In contrast to the first approach, pre-treatment of nutmeg extract decreased the frequency and duration of seizures as well as the duration of ictal discharges following PTZ injection. To elucidate the possible molecular mechanism of anti-seizure activity of nutmeg, hippocampal neuronal density was evaluated using histological staining. It has been well-documented that neuronal loss occurs in hippocampus of PTZ receiving animals (20, 21, 25). Our data suggests that the level of cell death was reduced in animals under pre-treatment of nutmeg extract. Furthermore, in agreement with previous studies, PTZ injection increased the level of glial activation in the hippocampus, and nutmeg extract administration ameliorated kindling-induced gliosis (19, 20, 26). 

Activated astrocytes and microglia secrete a variety of pro-inflammatory factors which actively participate in the progression of neurodegenerative diseases (27-29). Our data is consistent with previous reports which showed the beneficial anti-inflammatory effects of nutmeg extract, both *in-vitro *and *in-vivo*. In parallel with other studies, GC-MS analysis suggested that myristic acid, elemicin and myristicin are major chemical components of nutmeg extracts, which result in important anti-inflammatory effects (30). The anti-inflammatory effects of nonacetyleted fatty acids such as myristic acid has been well understood (31). It has been proposed that myristic acid exerts its anti-inflammatory action through inhibition of phospholipase A2 enzyme (32). Myristicin significantly ameliorates inflammation via its inhibitory role on production of nitric oxide (NO), cytokines, chemokine, and growth factors in dsRNA-stimulated macrophages (33). Furthermore, the anti-inflammatory effect of myristicin has been investigated in carrageenin-induced edema in rats and acetic acid-induced vascular permeability in mice. It was observed that the anti-inflammatory activity of myristicin was the same as indomethacin (14). Additionally, phenylpropanoids including elemicin and myristicin inhibit the production of IL-1β and IL-6 which lead to beneficial anti-inflammatory effects in lung inflammation (34). *Myristica fragrans* suppressed the production of NO and cyclooxygenase (COX-2) which are crucial inflammation mediators in lipopolysaccharide (LPS)-induced inflammation model (35). In addition, nutmeg oil can potentially alleviate the chronic inflammatory pain by inhibiting COX-2 expression and releasing substance P (15). Furthermore, other components of nutmeg extract, such as eugenol, also possess important anti-inflammatory activity. It has been well addressed that eugenol inhibits the production of pro-inflammatory cytokines and suppresses redox signaling pathway in LPS-induced lung inflammation (36). 

Overall, this study indicates that myristic acid, elemicin, and myriticin are the major components of ethanolic extract of nutmeg. Pre-treatment of nutmeg extract reduce seizure behavior in PTZ receiving animals. The anti-convulsant activity of nutmeg extract is partly mediated through the amelioration of glial activation. Furthermore, the level of hippocampal neuronal loss was alleviated in animals under treatment of nutmeg extracts. According to these findings, nutmeg extract might be useful as a natural supplementary compound in epileptic patients. 

## References

[1] Badawy RA, Harvey AS, Macdonell RA (2009). Cortical hyperexcitability and epileptogenesis: understanding the mechanisms of epilepsy - part 1. J.Clin.Neurosci.

[2] Vezzani A, French J, Bartfai T, Baram TZ (2011). The role of inflammation in epilepsy. Nat. Rev. Neurol.

[3] Vezzani A, Granata T (2005). Brain inflammation in epilepsy: experimental and clinical evidence. Epilepsia.

[4] Vezzani A, Aronica E, Mazarati A, Pittman QJ (2013). Epilepsy and brain inflammation. Exp. Neurol.

[5] Vezzani A, Balosso S, Ravizza T (2008). The role of cytokines in the pathophysiology of epilepsy. Brain. Behav. Immun.

[6] Jung KH1, Chu K, Lee ST, Kim J, Sinn DI, Kim JM, Park DK, Lee JJ, Kim SU, Kim M, Lee SK, Roh JK (2006). Cyclooxygenase-2 inhibitor, celecoxib, inhibits the altered hippocampal neurogenesis with attenuation of spontaneous recurrent seizures following pilocarpine-induced status epilepticus. Neurobiol. Dis.

[7] Somera-Molina KC, Nair S, Van Eldik LJ, Watterson DM, Wainwright MS (2009). Enhanced microglial activation and proinflammatory cytokine upregulation are linked to increased susceptibility to seizures and neurologic injury in a ′two-hit′ seizure model. Brain. Res.

[8] Schmidt D (2011). Efficacy of new antiepileptic drugs. Epilepsy Curr.

[9] Ortinski P, Meador KJ (2004). Cognitive side effects of antiepileptic drugs. Epilepsy. Behav.

[10] Nsour W, Lau C-S, Wong I (2000). Review on phytotherapy in epilepsy. Seizure.

[11] Nagja T, Vimal K andSanjeev A (2016). Myristica fragrans: a comprehensive review. Int. J. Pharm. Pharm. Sci.

[12] Gupta AD, Bansal VK, Babu V, Maithil N (2013). Chemistry, antioxidant and antimicrobial potential of nutmeg (Myristica fragrans Houtt). J. Genet. Eng. Biotechnol.

[13] Olaleye M, Akinmoladun C, Akindahunsi A (2006). Antioxidant properties of Myristica fragrans (Houtt) and its effect on selected organs of albino rats. Afr. J. Biotechnol.

[14] Ozaki Y, Soedigdo S, Wattimena YR, Suganda AG (1989). Antiinflammatory effect of mace, aril of Myristica fragrans Houtt and its active principles. Jpn. J. Pharmacol.

[15] Zhang WK, Tao SS, Li TT, Li YS, Li XJ, Tang HB, Cong RH, Li Ma F, Wan CJ (2016). Nutmeg oil alleviates chronic inflammatory pain through inhibition of COX-2 expression and substance P release in vivo. Food Nutr. Res.

[16] Lee JY, Park W (2011). Anti-inflammatory effect of myristicin on RAW 2647 macrophages stimulated with polyinosinic-polycytidylic acid. Molecules.

[17] Dhir A (2012). Pentylenetetrazol (PTZ) kindling model of epilepsy. Curr. Protoc. Neurosci.

[18] Naeimi R, Ghasemi-Kasman M, Kazemi S, Ashrafpour M, Moghadamnia AA, Pourabdolhossein F (2018). Zingiber officinale extract pre-treatment ameliorates astrocytes activation and enhances neuroprotection in pentylenetetrazol-induced kindling model of epilepsy in mice. Physiol. Pharmacol.

[19] Hashemian M, Anissian D, Ghasemi-Kasman M, Akbari A, Khalili-Fomeshi M, Ghasemi S, Ahmadi F, Moghadamnia AA, Ebrahimpour A (2017). Curcumin-loaded chitosan-alginate-STPP nanoparticles ameliorate memory deficits and reduce glial activation in pentylenetetrazol-induced kindling model of epilepsy. Prog. Neuropsychopharmacol. Biol. Psychiatry.

[20] Anissian D, Ghasemi-Kasman M, Khalili-Fomeshi M, Akbari A, Hashemian M, Kazemi S, Moghadamnia AA (2018). Piperine-loaded chitosan-STPP nanoparticles reduce neuronal loss and astrocytes activation in chemical kindling model of epilepsy. Int. J. Biol. Macromol.

[21] Gol M, Ghorbanian D, Hassanzadeh S, Javan M, Mirnajafi-Zadeh J, Ghasemi-Kasman M (2016). Fingolimod enhances myelin repair of hippocampus in pentylenetetrazol-induced kindling model. Eur. J. Pharm. Sci.

[22] Saha L, Bhandari S, Bhatia A, Banerjee D, Chakrabarti A (2014). Anti-kindling effect of bezafibrate, a peroxisome proliferator-activated receptors alpha agonist, in pentylenetetrazole induced kindling seizure model. J. Epilepsy Res.

[23] Sonavane G, Palekar R, Kasture V, Kasture S (2002). Anticonvulsant and behavioural actions of Myristica fragrans seeds. Indian. J. Pharmacol.

[24] Wahab A, Haq RU, Ahmed A, Khan RA, Raza M (2009). Anticonvulsant activities of nutmeg oil of Myristica fragrans. Phytother. Res.

[25] Naseer M, Ullah N, Ullah I, Koh P, Lee H, Park M, Kim MO (2011). Vitamin C protects against ethanol and PTZ‐induced apoptotic neurodegeneration in prenatal rat hippocampal neurons. Synapse.

[26] Zhu X, Dong J, Shen K, Bai Y, Zhang Y, Lv X, Chao J (2015). HNMDA receptor NR2B subunits contribute to PTZ-kindling-induced hippocampal astrocytosis and oxidative stress. Brain. Res. Bull.

[27] Ben Haim L, Carrillo-de Sauvage MA, Ceyzeriat K, Escartin C (2015). Elusive roles for reactive astrocytes in neurodegenerative diseases. Front. Cell Neurosci.

[28] Liu B, Hong JS (2003). Role of microglia in inflammation-mediated neurodegenerative diseases: mechanisms and strategies for therapeutic intervention. J. Pharmacol. Exp. Ther.

[29] Maragakis NJ, Rothstein JD (2006). Mechanisms of Disease: astrocytes in neurodegenerative disease. Nat. Clin. Pract. Neurol.

[30] Yang XW, Huang X, Ahmat M (2008). New neolignan from seed of Myristica fragrans. Zhongguo Zhong Yao Za Zhi.

[31] Sharma N, Samarakoon KW, Gyawali R, Park Y-H, Lee S-J, Oh SJ, Jeong DK (2014). Evaluation of the antioxidant, anti-inflammatory, and anticancer activities of Euphorbia hirta ethanolic extract. Molecules.

[32] Gopi K, Renu K, Jayaraman G (2014). Inhibition of Naja naja venom enzymes by the methanolic extract of Leucas aspera and its chemical profile by GC–MS. Toxicol. Rep.

[33] Lee JY, Park W (2011). Anti-inflammatory effect of myristicin on RAW 2647 macrophages stimulated with polyinosinic-polycytidylic acid. Molecules.

[34] Lim HJ, Woo KW, Lee KR, Lee SK, Kim HP (2014). Inhibition of Proinflammatory Cytokine Generation in Lung Inflammation by the Leaves of Perilla frutescens and Its Constituents. Biomol. Ther.

[35] Min BS, Cuong TD, Hung TM, Min BK, Shin BS, Woo MH (2011). Inhibitory Effect of Lignans from Myristica fragrans on LPS-induced NO Production in RAW2647 Cells B. Korean. Chem. Soc.

[36] Huang X, Liu Y, Lu Y, Ma C (2015). Anti-inflammatory effects of eugenol on lipopolysaccharide-induced inflammatory reaction in acute lung injury via regulating inflammation and redox status. Int. J. Immunopharmacol.

